# Pathways from developmental vulnerabilities in early childhood to schizotypy in middle childhood

**DOI:** 10.1111/bjc.12405

**Published:** 2022-12-02

**Authors:** Kirstie O'Hare, Oliver Watkeys, Johanna C. Badcock, Kristin R. Laurens, Stacy Tzoumakis, Kimberlie Dean, Felicity Harris, Vaughan J. Carr, Melissa J. Green

**Affiliations:** ^1^ Discipline of Psychiatry and Mental Health, School of Clinical Medicine University of New South Wales Sydney NSW Australia; ^2^ School of Psychological Science University of Western Australia Perth WA Australia; ^3^ School of Psychology and Counselling Queensland University of Technology (QUT) Brisbane Qld Australia; ^4^ School of Criminology and Criminal Justice Griffith University Southport Qld Australia; ^5^ Justice Health and Forensic Mental Health Network Sydney NSW Australia; ^6^ Neuroscience Research Australia Sydney NSW Australia; ^7^ Department of Psychiatry Monash University Melbourne Vic. Australia

**Keywords:** cognitive vulnerability, developmental antecedents, path analysis, schizophrenia‐spectrum disorders, schizotaxia, schizotypy

## Abstract

**Objectives:**

Childhood disturbances in social, emotional, language, motor and cognitive functioning, and schizotypy have each been implicated as precursors of schizophrenia‐spectrum disorders. We investigated whether relationships between early childhood developmental vulnerabilities and childhood schizotypy are mediated by educational underachievement in middle childhood.

**Methods:**

Participants were members of a large Australian (*n* = 19,216) population cohort followed longitudinally. Path analyses were used to model relationships between developmental vulnerabilities at age ~5 years, educational underachievement from ages ~8 to 10 years and three distinct profiles of schizotypy at age ~11 years (*true*, *introverted* and *affective schizotypy*).

**Results:**

Early childhood developmental vulnerabilities on five broad domains (related to physical, emotional, social, cognitive and communication development) were associated with schizotypy profiles in middle childhood. Educational underachievement in middle childhood was associated with all schizotypy profiles, but most strongly with the *true schizotypy* profile (OR = 3.92, 95% CI = 3.12, 4.91). The relationships between schizotypy profiles and early childhood developmental vulnerabilities in ‘language and cognitive skills (school‐based)’ and ‘communication skills and general knowledge’ domains were fully mediated by educational underachievement in middle childhood, and the relationships with early childhood ‘physical health and well‐being’ and ‘emotional maturity’ domains were partially mediated.

**Conclusion:**

Developmental continuity from early childhood developmental vulnerabilities to schizotypy in middle childhood is mediated by educational underachievement in middle childhood. While some domains of early developmental functioning showed differential relationships with distinct schizotypy profiles, these findings support a developmental pathway to schizotypy in which cognitive vulnerability operates from early childhood through to middle childhood.

**Practitioner Points**

- Children aged 11–12 years report characteristics of schizotypy that are associated with early childhood social, emotional, language and motor functioning, and cognitive vulnerability.
- Cognitive dysfunction at school entry, extending into middle childhood, may be part an important element of a developmental pathway of schizotypy.
- Distinct profiles of schizotypy were associated with different types of early childhood antecedents, suggesting there may be different developmental pathways leading to schizophrenia (and potentially other mental disorders).

## INTRODUCTION

Over the last 30 years, accumulating evidence has supported a neurodevelopmental model of schizophrenia, which posits that disruption in early brain development (observable as developmental abnormalities) underpins the development of schizophrenia in late adolescence or adulthood (Murray et al., 2017; Murray & Lewis, 1987; Weinberger, 1987). This idea of neurodevelopmental continuity between early childhood antecedents and later disorder aligns closely with models of schizotypy, a construct reflecting latent liability for schizophrenia (Claridge, 1997; Meehl, 1962, 1990). Meehl (1990) conceptualized schizotypy as a phenotypic expression of a neural diathesis present during postnatal brain development. This neural diathesis (*schizotaxia* or *cognitive slippage*) is thought to interact with other (e.g., environmental and psychosocial) risk factors across the lifespan, which, in extreme cases or in adverse environments, can result in the onset of clinical disorder. In this way, schizotypy can be viewed as an intermediate phenotype on the pathway to schizophrenia and provides a useful framework for understanding the developmental nature of schizophrenia (Debbané et al., 2015; Debbané & Barrantes‐Vidal, 2015).

There is extensive research indicating that children who go on to develop schizophrenia display subtle deficits (i.e., developmental vulnerabilities) in motor, social, emotional, cognitive and language functioning in early childhood long before overt onset of psychotic symptoms (for review, see Dickson et al., 2012; Laurens et al., 2015; Niemi et al., 2003; Rapoport et al., 2005; Welham, Isohanni, et al., 2009). These effects seem robust across a range of study designs, including behavioural and familial high‐risk studies (Niemi et al., 2003), as well as general population birth cohorts (Welham, Isohanni, et al., 2009). Of note, there is some evidence of sex differences, in that developmental vulnerabilities do not necessarily present at equivalent times or rates in boys and girls (Zambrana et al., 2012), and elevated levels of childhood vulnerabilities may predict different outcomes for girls versus boys (Dekker et al., 2007). Furthermore, social, emotional, cognitive and language impairments in early childhood are associated with polygenic risk for schizophrenia (Riglin et al., 2017), suggesting that these developmental impairments could be early manifestations of genetic risk. While the evidence for the relationship of early childhood developmental impairments with schizophrenia has been well established, it is still unclear which are specific to schizophrenia, and which confer liability to psychotic disorders (or other serious mental illnesses) more generally (Laurens et al., 2015). Furthermore, despite conceptualizations of schizotypy as inherently developmental in nature, there is little research examining associations of early childhood developmental impairments with later schizotypy.

There is some evidence that early childhood developmental impairments are associated with psychotic‐like experiences (PLEs, which represent the ‘reality‐distortion’ or so‐called ‘positive’ features of schizotypy) in middle childhood (spanning ages 6–12 years), as well as being associated with schizophrenia in adulthood. One study found that developmental impairments in motor, cognitive and emotional domains in early childhood predicted PLEs at age 11 years, and both the developmental impairments *and* PLEs predicted the diagnosis of schizophrenia by age 26 (Cannon et al., 2002). Furthermore, another birth cohort study found that emotional functioning at age 5 years was associated with both PLEs at age 14 and non‐affective psychotic disorder at age 21 (Welham, Scott, et al., 2009). These findings suggest that early childhood developmental impairments and schizotypy may both be manifestations of a single developmental process, rather than independent processes. That is, there is likely intra‐individual developmental continuity between early childhood developmental impairments, later schizotypy and subsequent schizophrenia‐spectrum disorders.

However, PLEs reflect just one aspect of schizotypy; the latter also includes features known as ‘negative schizotypy’, including variations in cognitive, interpersonal and affective functioning. Different aspects of schizotypy may not be uniformly related to early childhood developmental antecedents, as the substantial heterogeneity in schizophrenia and schizotypy may result from the presence of distinct subtypes with different developmental pathways (Crow, 1980; Jablensky, 2006; Meehl, 1990; Raine, 2006). That is, early childhood antecedents may be associated with the co‐occurrence of schizotypal features within an individual (i.e., person‐centred pattern) rather than dimensions of schizotypy that are distributed continuously within a population. One cross‐sectional study examined the association between distinct schizotypy clusters with neurocognitive, neurological and behavioural correlates (Barrantes‐Vidal et al., 2003). Four schizotypy clusters were found—*high positive schizotypy*, *high negative schizotypy*, *high on both positive and negative*, and *low on both*. The *high negative schizotypy* and *high on both* groups were associated with worse performance on the neurocognitive, behavioural and neurological measures, while the *high positive schizotypy* and *low on both* groups were not. This suggests possible specificity of the relationship between negative schizotypy and neurodevelopmental impairments. Interestingly, much of the research on subtyping schizophrenia has also focused on cognitive features as a target for distinguishing groups (Carpenter et al., 1988, 1999; Jablensky, 2006), with studies consistently identifying subtypes characterized by differences in cognitive functioning (Green et al., 2020). Recent genetic evidence corroborates the idea of cognitive subtypes of schizophrenia (with distinct genetic contributions; Bansal et al., 2018; Green et al., 2013; Hallmayer et al., 2005), such that cognitive functioning could be a key variable in explaining distinct pathways to different subtypes of both schizophrenia and schizotypy.

Cognitive deficits in schizophrenia are ubiquitous and considered core to the disorder (Kahn & Keefe, 2013). Individuals who go on to develop schizophrenia display subtle cognitive dysfunction in childhood, which then continues to lag behind typically developing children (Rapoport et al., 2005, 2012). A widening lag in cognitive development prior to psychosis seems specific to schizophrenia, in comparison with other severe mental illnesses (such as bipolar and major depressive disorders), where cognitive dysfunction may be present once the disorder fully developed, but not generally beforehand (Meier et al., 2014; Sheffield et al., 2018). Cognitive decline prior to psychosis may distinguish schizophrenia from other psychiatric disorders (Kahn & Keefe, 2013). Cognitive impairment is also found in schizotypy (Ettinger et al., 2015) and is consistent with Meehl's model of schizotypy where *schizotaxia* or *cognitive slippage* is considered a defining feature (Meehl, 1990). To our knowledge, no previous research has investigated how childhood cognitive dysfunction may operate within a developmental model of schizotypy.

The aim of the present study was to investigate the relationships between early childhood developmental vulnerabilities (age ~5 years), educational underachievement in middle childhood (as a measure of cognitive vulnerability, age 8–10 years), and three previously identified schizotypy profiles in middle childhood (age ~11 years; Green et al., 2022), using data drawn from a large (*n* ~19,000) cohort followed longitudinally within the New South Wales‐Child Developmental Study (NSW‐CDS). Early childhood developmental vulnerabilities included ‘physical health and well‐being’ (e.g., fine and gross motor skills), ‘social competence’ (e.g., social skills), ‘emotional maturity’ (e.g., aggression), ‘language and cognitive skills [school‐based]’ (e.g., literacy) and ‘communication skills and general knowledge’ (e.g., ability to communicate effectively). The three schizotypy profiles were characterized by (1) high levels of negative and cognitive features, labelled *true schizotypy*, (2) high levels of positive and affective features, labelled *affective schizotypy* and (3) moderate levels of negative and cognitive features with low levels of positive features, labelled *introverted schizotypy*. Specific aims were to investigate (1) whether distinct schizotypy profiles in middle childhood have differential relationships with early childhood developmental vulnerabilities; (2) whether relationships between early childhood developmental vulnerabilities and schizotypy in middle childhood are mediated by educational underachievement in middle childhood; and (3) whether these relationships differ in boys and girls.

## METHODS

### Study setting and record linkage

Data were obtained from Wave 2 of the NSW‐CDS, an Australian intergenerational, longitudinal, multi‐agency record linkage study (Carr et al., 2016; Green et al., 2018). In the NSW‐CDS, administrative record data is linked with two cross‐sectional surveys; the Australian Early Development Census (AEDC; Brinkman et al., 2014) collected at age ~5 years (in 2009), and the Middle Childhood Survey (MCS; Laurens et al., 2017) collected at age ~11 years (in 2015). In the current study, we used data from the AEDC and the MCS linked with data from the Australian Curriculum, Assessment and Reporting Authority's (ACARA) National Assessment Program (managed by the NSW Education Standards Authority). The NSW Centre for Health Record Linkage (CHeReL) conducted the record linkages, using probabilistic linkage methods spanning a set of personal identifiers, with an estimated false‐positive linkage rate of <.5% (Green et al., 2018). Ethics approval was obtained from the NSW Population and Health Research Ethics Committee (reference, HREC/15/CIPHS/21).

### Participants

Participants were 19,216 children who had complete data available on the AEDC and had schizotypy profile information from the MCS as per methods described in detail elsewhere (Green et al., 2022). Half of the children were female (50.6%; *n* = 9737), 6.9% (*n* = 1330) were of Aboriginal and/or Torres Strait Islander (Indigenous) descent, and 8.5% (*n* = 1640) had a language background other than English. These demographic characteristics of this subsample are comparable to the full MCS sample, which has been found to be similar to the broader NSW population (Laurens et al., 2017).

### Measures

#### Early childhood developmental vulnerabilities (age ~ 5 years)

Early childhood developmental vulnerabilities were measured using the AEDC, a 96‐item teacher‐rated measure collected for children during their first year of full‐time schooling (at age ~ 5 years). The AEDC measures developmental competencies in five broad domains, and children are considered *developmentally vulnerable* in a domain if they score in the below the 10th percentile of the national population distribution (Brinkman et al., 2014). The AEDC has satisfactory construct and concurrent validity, and the five domains have displayed good reliability (Table S1); and α range from .80 to .95; (Brinkman et al., 2007; Janus et al., 2011).

The five broad domains measured by the AEDC are:

1. *Physical health and well‐being*—fine and gross motor skills, physical readiness for the school day, physical independence, handedness and coordination.
2. *Social competence*—overall social skills, respect and responsibility towards others, ability to work independently and follow class routines, readiness to explore new things.
3. *Emotional maturity*—aggressiveness, hyperactivity and inattention, prosocial and helping behaviours, and anxious and fearful behaviours.
4. *Language and cognitive skills (school‐based)—*basic and advanced literacy and numeracy, interest in literacy and numeracy, and memory.
5. *Communication skills and general knowledge*—ability to communicate effectively, ability to participate in games involving language (including story‐telling and imaginative play), general knowledge about the world.

#### Sustained educational underachievement (age ~ 8–10 years)

Cognitive vulnerability in middle childhood was indexed using a measure of educational underachievement derived from data from the National Assessment Program—Literacy and Numeracy (NAPLAN) for children enrolled in grades 3 and 5 (ages ~8 and ~10 years, respectively). Children were categorized as having sustained educational underachievement if they failed to attain the specified national minimum standard in at least one of the five NAPLAN assessments (numeracy, reading, writing, spelling, or grammar and punctuation) in *both* grade 3 and grade 5. While NAPLAN is an assessment of academic achievement, performance on NAPLAN has previously been strongly linked to cognitive abilities (Carmichael et al., 2014).

#### Schizotypy (age ~11 years)

Schizotypy was assessed using self‐report data from the MCS. Three profiles of schizotypy were identified in a previous analysis of the NSW‐CDS data, using latent profile analysis of six domains of schizotypy (unusual experiences, cognitive disorganization, impulsive non‐conformity, introversion‐asociality, anxiety and depression, and self‐other disturbance), derived from 59 MCS items among a sample of *n* = 22,137 participants (Green et al., 2022). The six schizotypy domains display good reliability (Table S1). The profiles identified were characterized by: (1) high levels of cognitive disorganization, impulsive non‐conformity, introversion and self‐other disturbance, and low levels of unusual experiences—labelled *true schizotypy* (comprising 5.7% of the *n* = 19,216 sample), (2) high levels of unusual experiences and anxiety/depression—labelled *affective schizotypy* (18.8% of the sample) and (3) high introversion, moderate cognitive disorganization, impulsive non‐conformity and self‐other disturbances, and low levels of unusual experiences and anxiety/depression—labelled *introverted schizotypy* (19.9% of the sample); the rest of the sample (55.6%) displayed no signs of schizotypy and were labelled *no risk*.

### Data analysis

Tetrachoric correlations between key variables were calculated to check for collinearity between variables prior to path analyses (no correlations were > .85; Barbeau et al., 2019). Multinomial logistic regression analysis was used to estimate associations between schizotypy profile membership and (1) sustained educational underachievement and (2) the five AEDC domains. Then, binary logistic regression was used to estimate associations between sustained educational achievement and the five AEDC domains. Analyses resulted in odds ratios (ORs) with 95% confidence intervals (CIs) as measures of effect size, with ORs of 1.00–1.49 interpreted as small, 1.50–2.49 as medium, and 2.50 or more as large (Rosenthal, 1996). Distinctions between groups (in terms of their association with specific risk factors) were compared in terms of point estimates, and whether 95% confidence intervals were non‐overlapping as a means of indicating significant differences in point estimates (Cumming & Finch, 2005). All analyses were adjusted for sex, and for analyses with the AEDC domains, each domain was adjusted for the four other domains. Correlation and regression analyses were conducted using SPSS Version 26.

We then conducted path analyses of the relationships between early childhood developmental vulnerabilities, sustained educational underachievement and membership in the three schizotypy profiles. Path analysis is an extension of regression, that simultaneously estimates associations between multiple independent and dependent variables allowing for examination of direct and indirect effects within a priori models. Modelled associations were unidirectional based on the temporal ordering of the variables (i.e., earlier exposures predict later outcomes). We modelled direct effects of the five AEDC domains on the three schizotypy risk profiles, as well as indirect effects via sustained educational underachievement. Analyses were conducted first for the whole sample and then separately for males and females. All path models were fitted in Mplus version 7 and estimated by maximum likelihood. Bias‐corrected confidence intervals were estimated using bootstrapping methods (Preacher & Hayes, 2008), with 500 replicates. Effects were considered significant if the 95% confidence interval did not include 1.

## RESULTS

Descriptive information for key variables is summarized in Table 1, and correlations between key variables are displayed in Table 2.

**TABLE 1:** Prevalence of key variables

	Total *n* (%)	*n* females (%)	*n* males (%)
Total *n* children	19,216	9737	9479
Sustained educational underachievement	913 (4.9%)	340 (3.5%)	573 (6.2%)
Early childhood developmental vulnerabilities			
Physical health and well‐being	1383 (7.2%)	474 (4.9%)	909 (9.6%)
Social competence	1420 (7.4%)	457 (4.7%)	963 (10.2%)
Emotional maturity	1168 (6.1%)	270 (2.8%)	898 (9.5%)
Language and cognitive skills (school‐based)	794 (4.1%)	281 (2.9%)	513 (5.4%)
Communication skills and general knowledge	1344 (7.0%)	484 (5.0%)	860 (9.1%)
Schizotypy class			
Affective schizotypy	3609 (18.8%)	2120 (21.8%)	1489 (15.8%)
Introverted schizotypy	3826 (19.9%)	1338 (13.7%)	2488 (26.2%)
True schizotypy	1092 (5.7%)	440 (4.5%)	652 (6.9%)

**TABLE 2:** Tetrachoric correlations between variables (*n* = 19,216)

	1.	2.	3.	4.	5.	6.	7.
1. Male	–						
2. Sustained educational underachievement	.18	–					
3. Developmental vulnerability on physical health and well‐being	.22	.36	–				
4. Developmental vulnerability on social competence	.25	.38	.64	–			
5. Developmental vulnerability on emotional maturity	.36	.33	.55	.83	–		
6. Developmental vulnerability on language and cognitive skills (school‐based)	.18	.62	.58	.63	.49	–	
7. Developmental vulnerability on communication skills and general knowledge	.19	.45	.65	.66	.51	.72	–
8. Affective schizotypy	−.14	.08	.10	.09	.07	.09	.09
9. Introverted schizotypy	.23	.09	.06	.07	.10	.10	.10
10. True schizotypy	.10	.14	.12	.16	.15	.09	.09

*Note*: All correlations are significant at *p* < .001.

### Associations between early childhood developmental vulnerability and schizotypy

Developmental vulnerabilities in physical health and well‐being, social competence and emotional maturity were associated with all three schizotypy profiles in analyses adjusted for sex and the other AEDC domains, with small to medium‐sized effects (Table 3). Developmental vulnerability in language and cognitive skills (school‐based) was associated with the *introverted schizotypy* group only, and developmental vulnerability in communication skills and general knowledge was not significantly associated with any of the schizotypy groups.

**TABLE 3:** Adjusted multinomial logistic regression of early childhood developmental vulnerabilities (age ~ 5 years) and sustained educational underachievement (from age 8 to 10 years), on schizotypy profiles at age ~ 11 years, and binary logistic regression of sustained educational underachievement on early childhood developmental vulnerabilities (*n* = 19,216)

	Affective schizotypy		Introverted schizotypy		True schizotypy		Sustained educational underachievement	
	aOR	95% CI	aOR	95% CI	aOR	95% CI	aOR	95% CI
Sustained educational underachievement	2.58	(2.16, 3.07)	2.43	(2.05, 2.89)	3.92	(3.12, 4.92)	–	–
Physical health and well‐being	1.51	(1.29, 1.77)	1.22	(1.04, 1.43)	1.59	(1.26, 2.00)	1.43	(1.15, 1.78)
Social competence	1.39	(1.17, 1.65)	1.19	(1.00, 1.41)	1.75	(1.36, 2.25)	1.22	(.95, 1.57)
Emotional maturity	1.71	(1.43, 2.06)	1.65	(1.38, 1.97)	1.96	(1.52, 2.53)	1.40	(1.09, 1.80)
Language and cognitive skills (school‐based)	1.22	(.99, 1.50)	1.29	(1.05, 1.57)	1.16	(.85, 1.58)	7.08	(5.72, 8.78)
Communication skills and general knowledge	1.14	(.97, 1.35)	1.11	(.94, 1.30)	.96	(.74, 1.24)	1.97	(1.58, 2.45)

Abbreviations: Adjusted, adjusted for sex, and for age 5 developmental vulnerabilities in all four other domains; aOR, adjusted odds ratio; CI, confidence interval.

### Associations between early childhood developmental vulnerability and sustained educational underachievement

Developmental vulnerability in language and cognitive skills (school‐based) was associated with a sevenfold increase in likelihood of sustained educational underachievement in middle childhood. Developmental vulnerabilities in the domains of physical health and well‐being, social competence, emotional maturity and communication skills and general knowledge were associated with sustained educational underachievement with small‐ to medium‐sized effects.

### Associations between sustained educational underachievement and schizotypy

Sustained educational underachievement was associated with all three schizotypy profiles with large‐sized effects, with the largest association with the *true schizotypy* profile. The effect sizes for the relationships of sustained educational achievement and the *introverted* and *affective* schizotypy profiles were comparable (i.e., point estimates were similar and confidence intervals were overlapping).

### Path analysis

**FIGURE 1:**
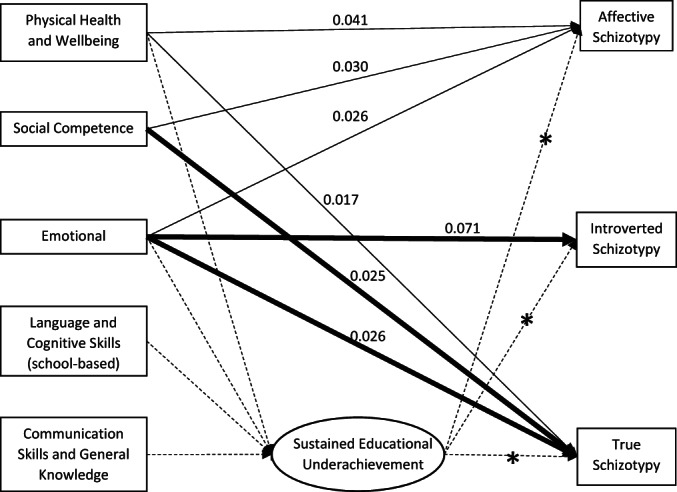
Direct and indirect effects of early childhood developmental vulnerabilities (age 5 years) on schizotypy profiles at age 11 years (only significant paths shown), for total sample (*n* = 19,216). *Note*: Bold line = medium effect, normal line = small effect. Solid lines indicate direct effects and dashed lines indicate indirect effects. *Line indicates the indirect effect of physical health and well‐being, emotional maturity, language and cognitive skills (school‐based), and communication skills and general knowledge domains.

Figure 1 describes the model in the whole sample (i.e., both boys and girls) with all significant pathways included (see also Table 4). Developmental vulnerability in physical health and well‐being, social competence and emotional maturity domains had direct effects on both *affective* and *true schizotypy* profiles. Additionally, developmental vulnerability in the emotional maturity domain had a direct effect on *introverted schizotypy*. Developmental vulnerability in the language and cognitive skills (school‐based), and communication skills and general knowledge domains did not have direct effects on any of the schizotypy profiles, but were both indirectly associated with all three schizotypy profiles via sustained educational underachievement. Physical health and well‐being and emotional maturity developmental vulnerabilities also had small indirect effects (via sustained educational underachievement) on all three schizotypy profiles.

**TABLE 4:** Direct and indirect effects of early childhood developmental vulnerabilities (age ~5 years) on schizotypy (age ~11 years), mediated by sustained educational underachievement in middle childhood (from age 8 to 10 years) for the total sample (*n* = 19,216)

	Direct effect		Indirect effect		Total effect	
	*b*	OR (95% CI)	*b*	OR (95% CI)	*b*	OR (95% CI)
Affective schizotypy						
Physical health and well‐being	**.041**	**1.295 (1.119, 1.485)**	**.001**	**1.005 (1.001, 1.012)**	**.042**	**1.302 (1.123, 1.491)**
Social competence	**.030**	**1.215 (1.035, 1.413)**	.000	1.003 (.999, 1.008)	**.031**	**1.218 (1.036, 1.417)**
Emotional maturity	**.026**	**1.182 (1.005, 1.391)**	**.001**	**1.006 (1.001, 1.013)**	**.027**	**1.189 (1.012, 1.395)**
Language and cognitive skills (school‐based)	−.001	.995 (.822, 1.213)	**.009**	**1.063 (1.030, 1.109)**	.008	1.057 (.874, 1.295)
Communication skills and general knowledge	.008	1.057 (.888, 1.237)	**.002**	**1.012 (1.005, 1.022)**	.010	1.069 (.899, 1.257)
Introverted schizotypy						
Physical health and well‐being	.011	1.075 (.934, 1.235)	**.001**	**1.007 (1.002, 1.014)**	.013	1.082 (.942, 1.244)
Social competence	.004	1.023 (.863, 1.202)	.001	1.003 (.999, 1.010)	.004	1.026 (.865, 1.207)
Emotional maturity	**.071**	**1.502 (1.271, 1.752)**	**.001**	**1.007 (1.002, 1.014)**	**.072**	**1.513 (1.281, 1.767)**
Language and cognitive skills (school‐based)	.013	1.087 (.891, 1.310)	**.012**	**1.077 (1.041, 1.123)**	.025	1.170 (.963, 1.401)
Communication skills and general knowledge	.013	1.085 (.913, 1.274)	**.002**	**1.014 (1.006, 1.025)**	.015	1.101 (.929, 1.290)
True schizotypy						
Physical health and well‐being	**.017**	**1.352 (1.072, 1.718)**	**.001**	**1.017 (1.004, 1.034)**	**.018**	**1.374 (1.096, 1.732)**
Social competence	**.025**	**1.539 (1.167, 1.981)**	.001	1.008 (.997, 1.025)	**.026**	**1.552 (1.171, 1.998)**
Emotional maturity	**.026**	**1.553 (1.219, 1.928)**	**.001**	**1.019 (1.005, 1.036)**	**.028**	**1.583 (1.262, 1.953)**
Language and cognitive Skills (school‐based)	−.010	.797 (.600, 1.097)	**.008**	**1.200 (1.124, 1.309)**	−.002	.956 (.720, 1.328)
Communication skills and general knowledge	−.006	.869 (.678, 1.092)	**.002**	**1.037 (1.017, 1.066)**	−.005	.902 (.702, 1.141)

*Note*: Bold indicates significant values (i.e., 95% CI does not include 1).

Abbreviations: *b*, parameter estimate; CI, confidence interval; OR, odds ratio.

Figure S1 describes the model in girls only (see also Table S2). In this model, physical health and well‐being, social competence and emotional maturity developmental vulnerabilities had direct effects on the *affective schizotypy* profile. Only developmental vulnerability in the physical health and well‐being domain had a direct effect on *true schizotypy*, and none of the AEDC domains had direct effects on *introverted schizotypy*. Developmental vulnerabilities in physical health and well‐being, emotional maturity, language and cognitive skills (school‐based), and communication skills and general knowledge domains had indirect effects via sustained educational underachievement on all three schizotypy profiles.

Figure S2 describes the model in boys only (see also Table S3). For boys, developmental vulnerability in the emotional maturity domain had a direct, small‐sized effect on all three schizotypy profiles. Additionally, developmental vulnerability in the physical health and well‐being domain had a direct effect on *affective schizotypy*, with a small‐sized effect, and developmental vulnerability in social competence had a medium‐sized direct effect on *true schizotypy*. Communication skills and general knowledge had a direct effect in the negative direction on *true schizotypy* (i.e., *not* being developmentally vulnerable in the communication skills and general knowledge domain was associated with membership in the *true schizotypy* profile). There were no direct effects of language and cognitive skills (school‐based) or communication skills and general knowledge domains, but both had indirect effects on all three schizotypy profiles via sustained educational underachievement.

## DISCUSSION

In a large, general population‐based cohort of Australian children, we found evidence that early childhood developmental vulnerabilities were associated with distinct schizotypy profiles in middle childhood. We also found that some of the associations between early childhood developmental vulnerabilities and schizotypy profiles were mediated by persistent educational underachievement in middle childhood (i.e., cognitive vulnerability measured at ages 8 and 10 years). Findings of differential relationships between the different domains of early childhood developmental vulnerabilities and the three distinct schizotypy profiles provide some support for the idea of subtypes of schizotypy in the general population having distinct developmental pathways. In particular, the relationship between early childhood developmental vulnerabilities in ‘language and cognitive skills (school‐based)’ and ‘communication skills and general knowledge’ and schizotypy was fully mediated by educational underachievement in middle childhood. These results suggest that schizotypy in childhood is part of a developmental pathway of cognitive vulnerability operating from early childhood to middle childhood. Lastly, analyses revealed differences in boys and girls, both in the rates of early childhood antecedents and the patterns of associations of these antecedents with schizotypy.

In analyses with the full sample, all five domains of early childhood developmental vulnerabilities (‘physical health and well‐being’, ‘social competence’, ‘emotional maturity’, ‘language and cognitive skills [school‐based]’ and ‘communication skills and general knowledge’) were associated with schizotypy in middle childhood, either directly or indirectly via mediation by educational underachievement in middle childhood. This adds to considerable evidence that early childhood developmental vulnerabilities represent one stage of a developmental pathway to schizophrenia (Dickson et al., 2012, 2020; Laurens et al., 2015; Niemi et al., 2003; Rapoport et al., 2005; Welham, Isohanni, et al., 2009) that accords with a developmental model of schizotypy. That is, these results support a model where developmental vulnerabilities and schizotypy are parts of a single developmental process, rather than independent processes. These findings also extend previous evidence for early childhood antecedents of PLEs at age 11 years (Cannon et al., 2002) by demonstrating associations between early childhood developmental vulnerabilities and broader, person‐centred, patterns of schizotypy in middle childhood. However, the present associations were mostly small, and the possibility of confounding by unmeasured variables (e.g., parenting factors) could not be ruled out.

The three distinct schizotypy profiles were not uniformly related to early childhood developmental vulnerabilities. The *true schizotypy* and *affective schizotypy* profiles had the most similar pattern of relationships, despite individuals in the *true schizotypy* profile displaying high levels of negative and cognitive features of schizotypy, while individuals in the *affective schizotypy* profile displaying high levels of positive features of schizotypy. Both the *true* and *affective schizotypy profiles* were associated with all five domains of early childhood developmental vulnerabilities, with direct effects evident for the physical health and well‐being, social competence and emotional maturity developmental vulnerabilities. By contrast, only the emotional maturity domain was directly associated with the *introverted schizotypy* profile (characterized by moderate levels of negative and cognitive features of schizotypy), along with small‐sized indirect effects of physical health and well‐being, emotional maturity, communication skills and general knowledge and language and cognitive skills (school‐based) domains via educational underachievement in middle childhood. These findings extend previous research showing that physical (Isohanni et al., 2001), emotional (Welham, Scott, et al., 2009), social (Tarbox & Pogue‐Geile, 2008), cognitive (Reichenberg et al., 2010) and language/communication (Bearden et al., 2000) deficits are independent predictors of later psychotic disorders, by showing that all five domains of developmental vulnerability predict at least some aspects of schizotypy when accounting for developmental vulnerability on the other domains. The findings that different types of developmental vulnerabilities in early childhood predict different profiles of schizotypy also provide some support for the idea that a number of different developmental pathways underpin the ‘group of schizophrenias’ (Bleuler, 1950; Carpenter et al., 1976) though differences in the associations between early childhood developmental vulnerabilities and schizotypy profiles were relatively subtle in terms of size (and differential patterns) of effects.

We found some evidence of a mediating role of educational underachievement in middle childhood in the developmental pathway from early childhood developmental vulnerabilities to schizotypy profiles at age ~ 11 years. The association between developmental vulnerabilities in language and cognitive skills (school‐based) and communication skills and general knowledge domains with all schizotypy profiles were fully mediated by educational underachievement in middle childhood, while developmental vulnerabilities in the physical health and well‐being and emotional maturity domains were only partially mediated by educational underachievement in middle childhood, albeit with a very small effect size. Interestingly, increased early childhood developmental vulnerability in social competence was *not* associated with educational underachievement in the full model. This may reflect that the measure of social competence was relatively broad in comparison with measures used in previous research on schizophrenia, which have typically focused on social withdrawal specifically. The association of developmental vulnerabilities in language and cognitive skills (school‐based) and communication skills and general knowledge domains with schizotypy is consistent with previous research, which has found that individuals who go on to develop schizophrenia display subtle cognitive and language deficits in childhood and adolescence (Bearden et al., 2000; Meier et al., 2014; Sheffield et al., 2018), and with conceptualizations of schizophrenia as a language disorder (Crow, 1997). The exact nature of developmental trajectories of cognition across individuals who go on to develop schizophrenia is unclear, mostly due to small sample sizes and short follow‐up times of previous research. The current findings suggest that subtle cognitive deficits may be apparent in individuals at risk of schizophrenia‐spectrum disorders as early as the time of school entry, and extending into middle childhood.

While all three schizotypy profiles were related to educational underachievement in middle childhood, the effect size for the relationship was largest (i.e., point estimate was highest and confidence intervals were non‐overlapping) for the *true schizotypy* profile, in comparison with the *affective* and *introverted schizotypy* profiles. It has been previously noted that the pattern of schizotypal characteristics displayed in the *true schizotypy* profile resembles Meehl's (1962, 1990) descriptions of the schizotaxia phenotype, while the *affective* and *introvertive* profiles more closely resemble Meehl's descriptions of *pseudo‐schizotypy* (Green et al., 2022). Meehl (1990) proposed that alongside a ‘true’ schizotypy phenotype—that was associated with genetically determined brain pathology (*schizotaxia* or *cognitive slippage*)—there may be other forms of *pseudo‐schizotypy*, in which an individual's personality and behavioural features appear phenotypically similar to schizotypy, but are the result of a combination of polygenically determined personality features interacting with traumatic events, rather than via *schizotaxia*. Along with evidence from more recent genetic studies which have provided support for the existence of high and low cognitive subtypes of schizophrenia (Bansal et al., 2018; Green et al., 2013; Hallmayer et al., 2005), it is plausible that there may be subtypes of schizotypy that are differentially related to cognitive functioning. While our findings provide some support for the idea that person‐centred patterns of schizotypal characteristics may be more or less related to cognitive vulnerability in early and middle childhood, all three schizotypy profiles were associated with sustained educational underachievement in middle school years, such that no single schizotypy profile represented a ‘high‐functioning’ or ‘non‐impaired’ cognitive subtype per se. These findings add weight to prior evidence of the benefit of investing in early education and brain development for long‐term well‐being (Australian Institute of Health and Welfare, 2015). Further analyses of educational achievement in later years of schooling may shed light on this issue, in later follow‐up of this population cohort.

Analyses conducted separately in boys and girls indicated some qualitative differences, consistent with previous research indicating that girls and boys may not present with developmental vulnerabilities at equivalent times or rates (Done et al., 1994; Isohanni et al., 2001; Laurens et al., 2007; Welham, Scott, et al., 2009). Interestingly, in girls, membership in the *true schizotypy* profile was associated with developmental vulnerability in the physical health and well‐being domain, whereas in boys, *true schizotypy* was associated with developmental vulnerabilities in emotional maturity and social competence domains. On average, girls and boys may reach developmental milestones at different rates (e.g., language; Zambrana et al., 2012), such that it is possible that these sex differences in associations between early childhood vulnerabilities may reflect that the cross‐sectional assessment completed at school entry is assessing a different stage of development for boys versus girls. Furthermore, there may be meaningful differences between boys and girls in developmental trajectories of emotional and behavioural functioning (e.g., emotional symptoms; Dekker et al., 2007), such that elevated levels of symptoms in childhood may predict different outcomes for boys versus girls.

Limitations of the study include that only cross‐sectional measures of developmental vulnerabilities (age ~ 5 years) and schizotypy (age ~ 11 years) were available, but these characteristics may not necessarily emerge only at these specific ages. Second, developmental vulnerabilities at age 5 years were measured by teacher report; while there is no ‘gold standard’ method (or optimal type of observer) to assess childhood behavioural and emotional characteristics, teachers will likely have different thresholds/perceptions of abnormal behaviour in children, compared with parents or clinicians (De Los Reyes & Kazdin, 2005). Third, we used a measure of sustained educational underachievement as a proxy index for poor cognitive function in middle childhood; however, educational achievement and cognitive functioning are overlapping but distinct constructs, subject to different genetic and environmental influences (Bartels et al., 2002; Davies et al., 2016; Thompson et al., 1991). Nevertheless, while educational achievement is an imperfect proxy for cognitive ability, it does measure a ‘real world’ application of a broad range of cognitive skills. Strengths of the study include the use of longitudinal data encompassing ~11 years of child development and the use of a variety of methods of prospective measurement (teacher report, administrative records and self‐report), which avoid interviewer and recall biases. Furthermore, we used a large sample that is broadly representative of the NSW population (Laurens et al., 2017), and used person‐centred analyses of patterns of schizotypal features which can better account for population heterogeneity in risk for schizophrenia than analyses of individual dimensions of schizotypy.

In conclusion, we report evidence that supports a model of developmental continuity from early and middle childhood cognitive vulnerability to schizotypy in middle childhood. There was some evidence of differing relationships between types of early childhood developmental vulnerability and distinct schizotypy profiles, but in general, differences were not substantial. We also found some evidence of mediation of this pathway by educational underachievement in middle childhood, especially for the *true schizotypy* profile, suggesting that cognitive impairment may be an important element of developmental pathways to schizophrenia‐spectrum disorders. Children with persistent cognitive vulnerabilities should therefore be prioritized for psychological support and prevention services. Future research is needed to fully examine the relationship of early childhood developmental vulnerabilities and schizotypy with mental health outcomes in adolescence and adulthood.

## AUTHOR CONTRIBUTIONS

**Kirstie O'Hare:** Formal analysis; methodology; writing – original draft. **Oliver Watkeys:** Formal analysis; methodology; writing – review and editing. **Johanna C. Badcock:** Funding acquisition; writing – review and editing. **Kristin R. Laurens:** Data curation; funding acquisition; project administration; writing – review and editing. **Stacy Tzoumakis:** Data curation; funding acquisition; project administration; writing – review and editing. **Kimberlie Dean:** Data curation; funding acquisition; project administration; writing – review and editing. **Felicity Harris:** Data curation; project administration; writing – review and editing. **Vaughan J. Carr:** Data curation; funding acquisition; writing – review and editing. **Melissa J. Green:** Conceptualization; data curation; funding acquisition; project administration; supervision; writing – review and editing.

## CONFLICT OF INTEREST

The authors declare that there are no conflict of interest to report.

## Supporting information


Appendix S1.

## Data Availability

The linked administrative data used in this study is owned by the Australian Government and cannot be made available to third parties by the authors.
