# Supplementary material for: Pathways from developmental vulnerabilities in early childhood to schizotypy in middle childhood
Source: Br J Clin Psychol. 2022 Dec 2;62(1):228–42. doi: 10.1111/bjc.12405 (PMC10946562; doi:10.1111/bjc.12405)
Supplement: Supplementary file 1 — Appendix S1. [file BJC-62-228-s001.docx]

**Pathways from early childhood developmental vulnerabilities to schizotypy in middle childhood**

SUPPLEMENTARY MATERIAL

**Table of Contents**

Supplementary Table 1. McDonald’s Omega reliability coefficients for each of the Australian Early Developmental Census (AEDC) and Schizotypy domain subscales, in the full sample (*n* = 19,216)...2

Supplementary Figure 1. Direct and indirect effects of early childhood developmental vulnerabilities (age ~5 years) on schizotypy profiles at age ~11 years (only significant paths shown), for girls only (n = 9,737)...............................................................................................................................................3

Supplementary Table 2. Direct and Indirect effects of early childhood developmental vulnerabilities (age ~5 years) on schizotypy (age ~11 years), mediated by sustained educational underachievement in middle childhood (from age 8 to 10), for girls only (n = 9,737).......................................................4

Supplementary Figure 2. Direct and indirect effects of early childhood developmental vulnerabilities (age ~5 years) on schizotypy profiles at age ~11 years (only significant paths shown), for boys only (n = 9,479)...............................................................................................................................................5

Supplementary Table 3. Direct and Indirect effects of early childhood developmental vulnerabilities (age ~5 years) on schizotypy (age ~11 years), mediated by sustained educational underachievement in middle childhood (from age 8 to 10 years), for boys only (n = 9,479) ............................................6

Supplementary Table 1. McDonald’s Omega reliability coefficients for each of the Australian Early Developmental Census (AEDC) and Schizotypy domain subscales, in the full sample (*n* = 19,216).

|  | **McDonald’s Omega** |
| --- | --- |
| **Australian Early Developmental Census (AEDC) Domains** |  |
| Physical Health and Wellbeing | 0.45 |
| Social Competence | 0.81 |
| Emotional Maturity | 0.66 |
| Language and Cognitive Skills (school-based) | 0.62 |
| Communication Skills and General Knowledge | 0.66 |
|  |  |
| **Schizotypy Domains** |  |
| Unusual Experiences | 0.83 |
| Cognitive Disorganisation | 0.79 |
| Impulsive Non-conformity | 0.81 |
| Introversion-Asociality | 0.77 |
| Anxiety and Depression | 0.78 |
| Self-other Disturbance | 0.86 |

Supplementary Figure 1. Direct and indirect effects of early childhood developmental vulnerabilities (age ~5 years) on schizotypy profiles at age ~11 years (only significant paths shown), for girls only (n = 9,737)

Sustained Educational Underachievement

Affective Schizotypy

Introverted Schizotypy

True Schizotypy

Physical Health and Wellbeing

Communication Skills and General Knowledge

Social Competence

Emotional Maturity

Language and Cognitive Skills (school-based)

*

*

*

*Note.* Bold line = medium effect, normal line = small effect. Solid lines indicate direct effects and dashed lines indicate indirect effects.

*Line indicates indirect effect of: physical health and wellbeing, emotional maturity, language and cognitive skills (school-based), and communication skills and general knowledge domains

Supplementary Table 2. Direct and Indirect effects of early childhood developmental vulnerabilities (age ~5 years) on schizotypy (age ~11 years), mediated by sustained educational underachievement in middle childhood (from age 8 to 10 years), for girls only (n = 9,737)

|  | Direct Effect | | Indirect Effect | | Total Effect | |
| --- | --- | --- | --- | --- | --- | --- |
|  | b | OR (95% CI) | b | OR (95% CI) | b | OR (95% CI) |
| *Affective Schizotypy* |  |  |  |  |  |  |
| Physical Health and Wellbeing | **0.054** | **1.347 (1.081, 1.666)** | **0.002** | **1.010 (1.001, 1.026)** | **0.055** | **1.361 (1.091, 1.679)** |
| Social Competence | **0.045** | **1.288 (1.004, 1.595)** | 0.000 | 1.003 (0.996, 1.012) | **0.045** | **1.292 (1.007, 1.666)** |
| Emotional Maturity | **0.064** | **1.424 (1.024, 1.899)** | **0.002** | **1.010 (1.000, 1.030)** | **0.066** | **1.438 (1.043, 1.922)** |
| Language and Cognitive Skills (school-based) | 0.001 | 1.006 (0.740, 1.334) | **0.012** | **1.072 (1.023, 1.137)** | 0.013 | 1.079 (0.802, 1.406) |
| Communication Skills and General Knowledge | 0.017 | 1.106 (0.873, 1.403) | **0.002** | **1.012 (1.002, 1.029)** | 0.019 | 1.119 (0.878, 1.417) |
|  |  |  |  |  |  |  |
| *Introverted Schizotypy* |  |  |  |  |  |  |
| Physical Health and Wellbeing | -0.009 | 0.927 (0.692, 1.242) | **0.002** | **1.014 (1.002, 1.033)** | -0.007 | 0.940 (0.701, 1.253) |
| Social Competence | 0.020 | 1.176 (0.821, 1.581) | 0.000 | 1.003 (0.995, 1.014) | 0.020 | 1.180 (0.827, 1.582) |
| Emotional Maturity | 0.041 | 1.373 (0.983, 1.907) | **0.002** | **1.013 (1.001, 1.034)** | 0.042 | 1.391 (0.995, 1.811) |
| Language and Cognitive Skills (school-based) | 0.023 | 1.204 (0.819, 1.698) | **0.013** | **1.096 (1.031, 1.183)** | 0.036 | 1.320 (0.903, 1.823) |
| Communication Skills and General Knowledge | -0.003 | 0.977 (0.732, 1.311) | **0.002** | **1.016 (1.003, 1.037)** | -0.001 | 0.993 (0.744, 1.325) |
|  |  |  |  |  |  |  |
| *True Schizotypy* |  |  |  |  |  |  |
| Physical Health and Wellbeing | **0.029** | **1.755 (1.176, 2.404)** | **0.002** | **1.027 (1.004, 1.057)** | **0.031** | **1.802 (1.216, 2.487)** |
| Social Competence | 0.005 | 1.129 (0.680, 1.754) | 0.000 | 1.007 (0.990, 1.034) | 0.005 | 1.137 (0.679, 1.766) |
| Emotional Maturity | 0.016 | 1.399 (0.781, 2.312) | **0.001** | **1.026 (1.002, 1.077)** | 0.017 | 1.435 (0.821, 2.327) |
| Language and Cognitive Skills (school-based) | -0.012 | 0.693 (0.336, 1.255) | **0.005** | **1.194 (1.063, 1.363)** | -0.007 | 0.827 (0.422, 1.474) |
| Communication Skills and General Knowledge | 0.015 | 1.326 (0.890, 2.020) | **0.002** | **1.032 (1.005, 1.079)** | 0.013 | 1.368 (0.909, 2.079) |

*Note.* Bold indicates significant values (i.e., 95% CI doesn’t include 1). b = parameter estimate; OR = odds ratio; CI = confidence interval.

Supplementary Figure 2. Direct and indirect effects of early childhood developmental vulnerabilities (age ~5 years) on schizotypy at age ~11 years (only significant paths shown), for boys only (n = 9,479)

Sustained Educational Underachievement

Affective Schizotypy

Introverted Schizotypy

True Schizotypy

Physical Health and Wellbeing

Communication Skills and General Knowledge

Social Competence

Emotional Maturity

Language and Cognitive Skills (school-based)

*Note.* Bold line = medium effect, normal line = small effect. Solid lines indicate direct effects and dashed lines indicate indirect effects.

Supplementary Table 3. Direct and Indirect effects of early childhood developmental vulnerabilities (age ~5 years) on schizotypy (age ~11 years), mediated by sustained educational underachievement in middle childhood (from age 8 to 10 years), for boys only (n = 9,479)

|  | Direct Effect | | Indirect Effect | | Total Effect | |
| --- | --- | --- | --- | --- | --- | --- |
|  | b | OR (95% CI) | b | OR (95% CI) | b | OR (95% CI) |
| *Affective Schizotypy* |  |  |  |  |  |  |
| Physical Health and Wellbeing | **0.040** | **1.340 (1.108, 1.611)** | 0.001 | 1.004 (0.998, 1.011) | **0.041** | **1.345 (1.116, 1.627)** |
| Social Competence | 0.024 | 1.197 (0.972, 1.516) | 0.000 | 1.003 (0.998, 1.012) | 0.024 | 1.200 (0.975, 1.530) |
| Emotional Maturity | **0.035** | **1.293 (1.042, 1.556)** | 0.001 | 1.005 (1.000, 1.015) | **0.036** | **1.300 (1.045, 1.564)** |
| Language and Cognitive Skills (school-based) | -0.001 | 0.996 (0.749, 1.250) | **0.009** | **1.072 (1.024, 1.139)** | 0.008 | 1.067 (0.818, 1.337) |
| Communication Skills and General Knowledge | 0.009 | 1.071 (0.851, 1.364) | **0.002** | **1.015 (1.004, 1.032)** | 0.011 | 1.087 (0.865, 1.383) |
|  |  |  |  |  |  |  |
| *Introverted Schizotypy* |  |  |  |  |  |  |
| Physical Health and Wellbeing | 0.008 | 1.045 (0.885, 1.250) | 0.001 | 1.003 (0.999, 1.008) | 0.009 | 1.048 (0.885, 1.249) |
| Social Competence | -0.011 | 0.942 (0.772, 1.155) | 0.000 | 1.002 (0.998, 1.009) | -0.011 | 0.944 (0.773, 1.157) |
| Emotional Maturity | **0.049** | **1.278 (1.057, 1.550)** | 0.001 | 1.004 (1.000, 1.010) | **0.050** | **1.283 (1.062, 1.555)** |
| Language and Cognitive Skills (school-based) | 0.007 | 1.035 (0.803, 1.302) | **0.010** | **1.054 (1.011, 1.102)** | 0.017 | 1.091 (0.865, 1.352) |
| Communication Skills and General Knowledge | 0.014 | 1.076 (0.887, 1.270) | **0.002** | **1.011 (1.002, 1.023)** | 0.016 | 1.088 (0.899, 1.285) |
|  |  |  |  |  |  |  |
| *True Schizotypy* |  |  |  |  |  |  |
| Physical Health and Wellbeing | 0.010 | 1.178 (0.906, 1.523) | 0.001 | 1.010 (0.995, 1.028) | 0.011 | 1.190 (0.919, 1.548) |
| Social Competence | **0.039** | **1.702 (1.255, 2.219)** | 0.001 | 1.008 (0.995, 1.030) | **0.040** | **1.717 (1.265, 2.214)** |
| Emotional Maturity | **0.026** | **1.467 (1.096, 1.969)** | 0.001 | 1.013 (0.999, 1.035) | **0.027** | **1.487 (1.120, 2.000)** |
| Language and Cognitive Skills (school-based) | -0.009 | 0.846 (0.579, 1.156) | **0.010** | **1.197 (1.099, 1.319)** | 0.001 | 1.012 (0.702, 1.366) |
| Communication Skills and General Knowledge | **-0.018** | **0.694 (0.495, 0.914)** | **0.002** | **1.040 (1.014, 1.085)** | **-0.016** | **0.722 (0.521, 0.965)** |

*Note.* Bold indicates significant values (i.e., 95% CI doesn’t include 1). b = parameter estimate; OR = odds ratio; CI = confidence interval.
